# Perception of COVID-19 Vaccination Amongst Physicians in Colombia

**DOI:** 10.3390/vaccines9030287

**Published:** 2021-03-19

**Authors:** Jorge L. Alvarado-Socarras, Andrea Liliana Vesga-Varela, Doris Cristina Quintero-Lesmes, Marcela M. Fama-Pereira, Norma C. Serrano-Diaz, Mauricio Vasco, Virgil Carballo-Zarate, Lysien I. Zambrano, Alberto Paniz-Mondolfi, Alfonso J. Rodriguez-Morales

**Affiliations:** 1 Fundación Cardiovascular de Colombia, Calle 155A No. 23–58, Floridablanca 681003, Colombia; jorgealvarado@fcv.org (J.L.A.-S.); dorisquintero@fcv.org (D.C.Q.-L.); normaserrano@fcv.org (N.C.S.-D.); 2 University of São Paulo, R. da Reitoria, 374–Cidade Universitária, Butantã, São Paulo 3550308, SP, Brazil; alilianavesga@usp.br; 3 Hospital Departamental del Quindio, San Juan de Dios, Universidad del Quindio, Armenia, Quindio 630004, Colombia; mfama@uniquindio.edu.co; 4 Sociedad Colombiana de Anestesiología y Reanimación, Bogotá 110911, Colombia; presidentescare@scare.org.co; 5 Asociación Colombiana de Medicina Interna, Bogotá 110911, Colombia; vircaza@hotmail.com; 6 Unit of Scientific Research, School of Medicine, Faculty of Medical Sciences, Universidad Nacional Autónoma de Honduras (UNAH), Tegucigalpa 11101, Honduras; lysien.zambrano@unah.edu.hn; 7 Latin American Network of Coronavirus Disease 2019 Research (LANCOVID), Pereira, Risaralda 660003, Colombia; Alberto.Paniz-mondolfi@mountsinai.org; 8 Laboratory of Microbiology, Department of Pathology, Molecular and Cell-Based Medicine, Icahn School of Medicine at Mount Sinai, New York, NY 10029-6574, USA; 9 Institutode Investigaciones Biomédicas IDB/Incubadora Venezolana de la Ciencia, Barquisimeto 3001, Venezuela; 10 Grupo de Investigación Biomedicina, Faculty of Medicine, Fundación Universitaria Autónoma de las Americas, Pereira, Risaralda 660003, Colombia; 11 School of Medicine, Universidad Privada Franz Tamayo, Cochabamba 4780, Bolivia

**Keywords:** COVID-19, SARS-CoV-2, pandemic, medical staff, vaccine

## Abstract

*Introduction*: The SARS-CoV-2/COVID-19 pandemic has triggered the need to develop rapidly effective and safe vaccines to prevent infection, particularly in those at-risk populations such as medical personnel. This study’s objective was to assess the perception of COVID-19 vaccination amongst Colombian physicians featuring two different scenarios of COVID-19 vaccination. *Methods*: A cross-sectional analytical study was carried out through an online survey directed at medical staff in several cities in Colombia. The percentage of physicians who have a positive perception to be vaccinated and the associated factors that determine that decision were determined. A binomial regression analysis adjusted for age and sex was carried out, taking as a dependent variable the acceptance of free vaccination with an effectiveness of 60 and 80%. The most significant factors were determined in the non-acceptance of vaccination. *Results*: Between 77.0% and 90.7% of physicians in Colombia accept COVID-19 vaccination, according to the scenario evaluated where the vaccine’s effectiveness was 60 or 80%, respectively. Medical specialty, having never paid for a vaccine, recommending the administration of the vaccine to their parents or people over 70 years, and dispensing the vaccine to their children, were the factors to consider to be vaccinated for free with an effectiveness of 60% and 80%. *Conclusions*: There is a high perception of the intention to vaccinate physicians in Colombia against COVID-19, and this is very similar to that of the general population.

## 1. Introduction

The coronavirus disease 2019 is the name assigned to the pathology caused by infection with the Severe Respiratory Acute Syndrome 2 (SARS-CoV-2) Coronavirus; initially reported in Wuhan, Hubei province, China, in December 2019. Due to its rapid worldwide distribution, the World Health Organization (WHO) declared it a pandemic in 2020 [1]. Although it displays a wide-age distribution, some groups are at higher risk for severe illness and death, such as the elderly (>70 years), people with comorbidities such as diabetes, hypertension, cardiovascular disease, and obesity. Additionally, other age groups have shown to be particularly susceptible, as is the case of children, young adults, pregnant women, who can also present unfavorable disease complications [2]. SARS-CoV-2 is an airborne infection causing a significant respiratory impact, leading to the rapid development of hypoxemia and death in at-risk populations [3].

Therefore, prevention measures, such as hand washing, social distancing (quarantines), and personal protection elements according to the spaces where people carry out their daily activities [4], are essential measures to tackle viral transmission. Due to the significant stress, the pandemic has posed risk on many levels, and control measures have been prioritized. However, some of these preventive measures, seeking to mitigate the virus’s spread, have proved largely ineffective, raising concerns about causing an economic crisis or secondary problems of confinement [5]. Strategies have also been designed to support the most seriously ill and to prevent deaths. Some interventions such as hydroxychloroquine, antivirals, macrolides, convalescent plasma, and steroids have not significantly impacted mortality reduction [6]. To date, there is no specific treatment for SARS-CoV-2 infection, which continues circulating widely while threatening to become endemic.

Efforts have not ceased to provide optimal care and treatment for those seriously ill and prevent disease progression. In this last scenario, vaccines play an important role [7]. The WHO has foreseen vaccination as the ultimate strategy to protect the most vulnerable. Physicians and other healthcare workers are included in this population, given their permanent risk for exposure despite utilization protective measures [8]. However, some studies exploring the “intention to vaccinate” in the general population have raised concerns that not all groups will accept receiving vaccination once widely available [9,10]. Undoubtedly, vaccination appears to be the best option to halt this pandemic, with health personnel being one of the priority groups. Caring for physicians and other frontline workers is a crucial step during the pandemic, generating greater confidence when caring for others, reducing the fear of being affected by the disease, and avoiding transmission to other family members [11]. As vaccination may prevent COVID-19 related deaths, case severity, hospitalizations, and transmission, it is key to have a high level of acceptance on currently available vaccine formulations, not only globally but in each specific country. Presently in Colombia, the Pfizer-BioNTech Vaccine (Comirnaty, also known as tozinameran or BNT162b2), and the Sinovac one (CoronaVac, formerly PiCoVacc) have become available, with other vaccines under development trusting to become available soon. So far, all of the approved vaccines, as well as those on early trials or limited used, have already shown an acceptable degree of efficacy and safety profile.

Finally, although the intention to be vaccinated in the general population is widely recognized, a recent survey of the National Department of Statistics revealed data from a national survey in 24 cities, ranging from 42.2% (Cali) to 72.5% (Riohacha) (https://www.dane.gov.co/index.php/estadisticas-por-tema/encuesta-pulso-social) (accessed on 1 March 2021); physicians’ acceptance remains unknown. Therefore, this work’s objective was to determine the perception of COVID-19 vaccination by physicians in Colombia with two different scenarios of the COVID-19 vaccine.

## 2. Material and Methods

A descriptive-analytical cross-sectional study was carried out. Data was collected from the self-completion of an electronic survey directed to physicians from different specialties in different Colombia cities in January 2021.

The survey was created on the Google forms platform. Dissemination of the survey was carried out by sending a link to the different medical societies, which were in charge of sending it to their fellow members according to each of their databases.

Once the survey was voluntarily self-completed, each of the participants’ information was uploaded into an Excel file, to which only the leading researcher of the study and the data analyst had access. For the statistical analysis, a descriptive analysis was initially performed, where the categorical values are presented as proportions and the continuous variables as means and standard deviation (SD). A bivariate analysis where two scenarios of possible vaccines were established generating two dependent variables: (a) “agree to apply a free vaccine with 60% effectiveness” and, (b) “accept to apply a free vaccine with 80% effectiveness”; and independent of all variables in the survey. The variables that obtained a *p* < 0.20 in the bivariate analysis were maintained in the multivariate models. All *p* values were taken in two tails, considering *p* < 0.05 as statistical significance. The association between the dependent variables (accepting to be vaccinated for free with a 60% and 80% effective vaccine) and independent variables (other variables) in this study was evaluated using binomial regression models with their corresponding goodness of fit evaluation. All data were analyzed using Stata^®^ version 14.0 statistical software (Stata Corporation, College Station, TX, USA).

Additionally, a theoretical relationship of the study variables was established. To represent the theoretical association between the intention to get vaccinated and the medical specialty, having paid for a vaccine, living with people over 70 years of age, giving the vaccine to their children, and recommending vaccination to parents or those over 70 years of age, adjusting for potentially confusing variables. The directed acyclic diagram (DAG) is a graphical tool used to represent a priori assumptions about the qualitative causal structure of the variables involved around a research question. The graph makes it possible to reveal systematic bias sources and identify possible design and analysis problems in the study [12].

### Ethical Considerations

The study was conducted under the Declaration of Helsinki. This research’s preparation and execution fully complied with the fundamental ethical principles of autonomy, justice, beneficence, and non-maleficence. The ethical approaches outlined in the code of medical ethics (Law 23 of 1981) and resolution 8430 of 1993 of the Ministry of Health of Colombia were complied with, which establish the standards for health research in which they participate. Humans. The Cardiovascular Research Foundation Colombia ethics committee approved it in Act No. 511, meeting on 25 August 2020.

## 3. Results

A total of 1066 surveys were completed and analyzed. Twenty-nine surveys were excluded since they did not match physicians with any specialty. Physicians answered 46.3% of the surveys. Departments such as Santander (11.9%) and Antioquia (10.8%) were the ones with the highest response, as well as the city of Bogotá (29.2%), which had the highest proportion of participation. Table 1 describes the population’s characteristics according to the acceptance of free vaccination with an effectiveness of 60% and 80%, respectively.

**Figure 1:**
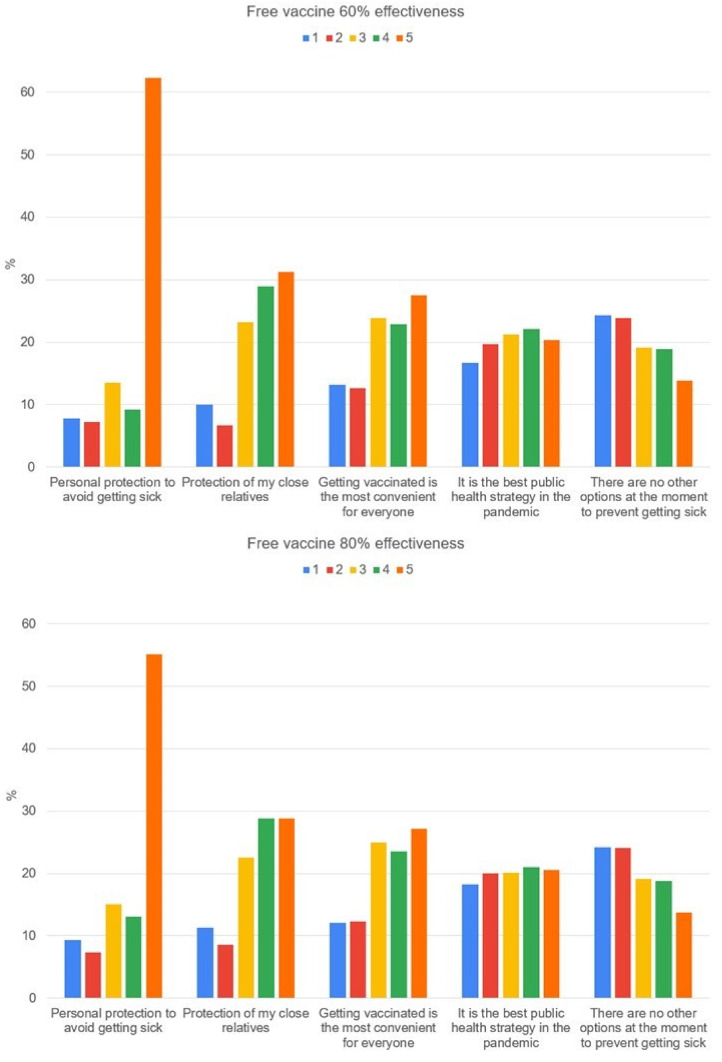
The reason why the participant would accept a free vaccination with an effectiveness of 60% and 80%, against COVID-19, one (1) being the least important and five (5) being the most important (2-4) intermediate choices.

**Figure 2:**
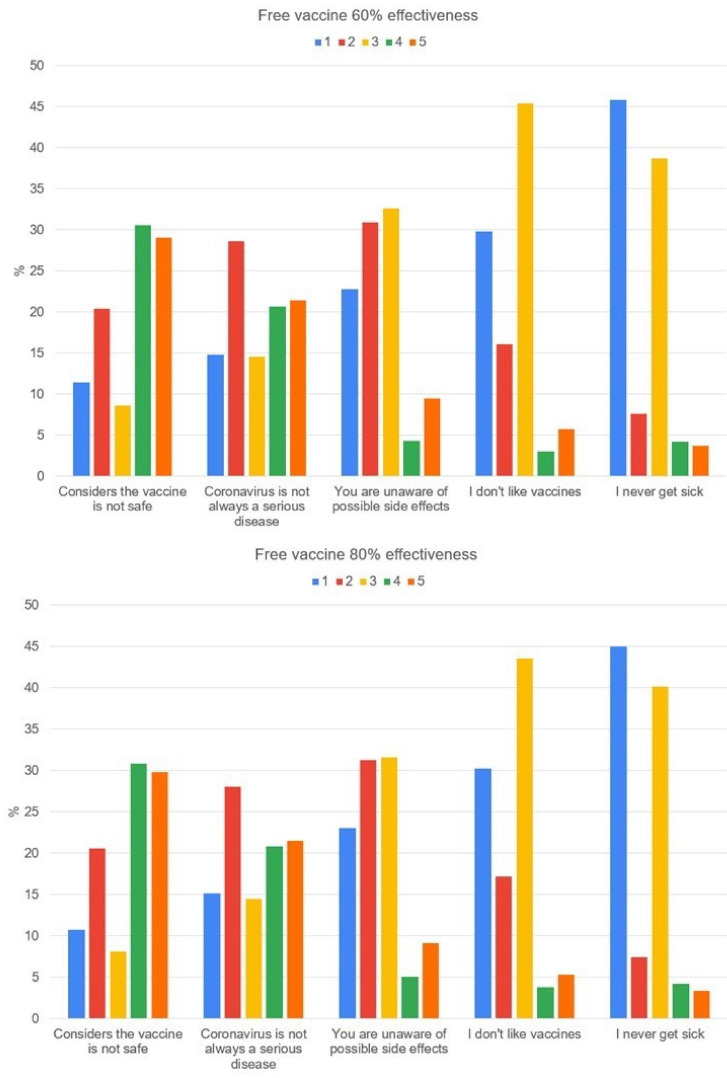
The reason why the participant would not accept a free vaccination with an effectiveness of 60% and 80%, against COVID-19, one (1) being the least important and five (5) the most important (2–4) intermediate choices.

Figure 1 shows why a participant would accept free vaccination with an effectiveness of 60% and 80%, against COVID-19, with one (1) being the least important and five (5) the most important. Figure 2 shows why the participant would not accept free vaccination with an effectiveness of 60% and 80%, against COVID-19, with one (1) being the least important and five (5) the most important.

Table 2 and Table 3 display the variables associated with accepting being vaccinated for free with an effectiveness of 60% and 80%, respectively, finding the same variables except for 60% of the department where they currently work and were related to the number of children.

**Figure 3:**
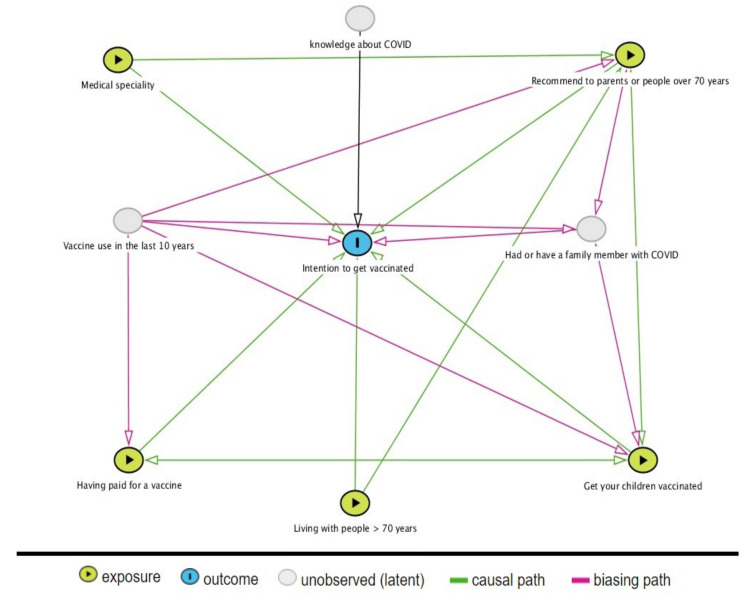
A causal diagram to represent the association between the intention to get vaccinated and the medical specialty, having paid for a vaccine, living with people over 70 years of age, giving their children the vaccine, and recommending vaccination to parents or those over 70 years of age, adjusting for potentially confusing variables. Source: Own elaboration through the website: http://www.dagitty.net/development/dags.html (accessed on 1 February 2021).

A directed acyclic diagram (DAG) was crafted and depicted in Figure 3. The following DAG shows two ways by which two types of bias could occur: the selection bias since the study is conditioned by the response of the surviving population and the residual confounding bias due to the non-adjustment for variables that were not included in the survey, such as the use of vaccines, having or having had a family member with COVID-19 (Figure 3). The testable implications were all assessed, showing that the data did not contradict the theoretical model.

## 4. Discussion

On 17 February 2021, the national COVID-19 vaccination plan started in Colombia, with the arrival, on previous days, of the first doses of the Pfizer-BioNTech (New York, NY, USA) COVID-19 vaccine (BNT162b2). Nevertheless, so far, up to 15 March 2021, only 913,961 doses have been applied, mainly in healthcare workers and the population ≥80-year-old. Colombia has a population of 51,049,498 people, with approximately 1.8 physicians per 1000 people (2021).

According to our findings, between 77.0 and 90.7% of screened physicians in Colombia would accept vaccination against COVID-19 in scenarios with a vaccine efficacy of 60 and 80%, respectively. Currently, the available vaccines, Pfizer-BioNTech (BNT162b2) and Sinovac (Beijing, China) (Coronavac) have reported efficacies of 95% and 50%. Then, given this, the refusal of scheduled physicians for any of these vaccines would be interesting to be assessed in the near future in the country. All the front-line healthcare workers are listed to be vaccinated in the first step of phase 1 of the national vaccination plan. Unfortunately, rumors on lack of interest in the application of the Sinovac among them, are currently circulating in the country. Then more medical education on the WHO criteria for approval and recommendation of COVID-19 vaccines, that include an efficacy that should be at least 50% is important (https://www.who.int/medicines/regulation/prequalification/prequal-vaccines/WHO_Evaluation_Covid_Vaccine.pdf?ua=1) (accessed on 1 March 2021) Moreover, it is worth mentioning that the efficacy for other endpoints, such as hospitalization, severe disease, and deaths, are higher than those mentioned.

Few studies have explored physicians’ intention to get vaccinated at the time of a broad commercial availability for the vaccine. A study amongst health workers in the Republic of Congo reported that only 27.7% of health workers would agree to be vaccinated [13]. However, there is some variability in this trend. In France, a study showed that 76.9% of health personnel would accept COVID-19 vaccination, and here in Colombia, this figure reached 90.7%. This work shows that physicians are the most inclined to receive the vaccination. Some factors associated with this positive intent included age (older age plus intention), gender, fear of COVID-19, individual risk perception [14]. This difference is likely associated with a better disease knowledge of the medical personnel regarding the benefits of vaccination and its impact where the surveys were conducted [15].

On the other hand, false information circulating on social media and other networks is likely a determining factor influencing vaccination in some groups [16]. In our case, it is unlikely that social networks have influenced the perception that medical personnel would have when it comes to getting vaccinated, as demonstrated by our results. Factors such as confidence or acceptance in scientific research and the efficacy of the vaccine are critical factors in the intention to be vaccinated [17]. When exploring the main reason for accepting vaccination in our work, self-protection was the main reason, as confirmed in previous work [14]. On the other hand, when exploring why they would not accept vaccination, the main factor was that they did not consider the vaccine safe. Very similar findings have been found in the general population about vaccination against H1N1 influenza [18], as well as in COVID-19 [19]. In a future investigation in Colombia, it would be interesting to compare the perceptions against vaccination between Influenza and COVID-19 among healthcare workers.

Other factors were also found to influence the intention to get vaccinated amongst medical staff; the main one was having ever paid for a vaccine. Additionally, working in hospitalization wards was associated with increased acceptance for vaccination. Although there is scarce data regarding this variable, physicians who have repeated contact with COVID 19 patients would have a greater risk of infection and, therefore, a greater intention to get vaccinated [20]. Data from India reveal that 75% of physicians affected by COVID-19 were over 50 years old [14,20]. However, specialists such as anesthesiologists, otolaryngology, and intensivists, who are also in close contact with infected patients, had increased acceptance to be vaccinated. However, our data failed to confirm the abovementioned. Although some factors were not significantly associated with intention to be vaccinated, likely, age, contact with people who had the disease, the number of people with whom one lives, living with people, or having some comorbidities are more significant relationship to get vaccinated.

Determining acceptance of vaccination within the medical and healthcare workers group is crucial to prevent the community’s misperceptions and potential rejection of vaccination against COVID-19 during the ongoing pandemic [21]. Community studies in countries such as Denmark, France, Germany, Italy, Portugal, Holland, the United Kingdom, and Australia have shown the population’s wide acceptance of the vaccine ranging between 73.9% and 85.7% [10,19]. The acceptance rate in the United States is around 70%. However, the same positive perception is particularly highlighted towards vaccine acceptance from the medical community, leading to public reassurance and non-rejection. It is recognized that in recent epidemics such as H1N1, the intention to be vaccinated has ranged from 50-to-64% [22]. However, this does not represent the magnitude of the current situation. Vaccination is the primary option for disease prevention and control, even though not accepted by all, including some physicians.

On the other hand, an additional factor for accepting COVID-19 vaccination relates to the previous history of vaccination against influenza [14]. However, it has been reported that acceptance would be much lower for influenza reports. Besides, some studies show that nursing personnel tends to be vaccinated than physicians, which may correlate to the disease’s knowledge [13,14,23].

Another interesting finding of the study is the relationship in recommending vaccination in potential risk populations such as children and adults over 70 years of age and the intention to be vaccinated in any evaluated scenario. Previous reports reveal similar caregivers’ findings, even accepting less rigorous processes in the vaccines’ development [24]. This can be explained by the fact that it is more feasible to recommend a vaccine if one is willing to use it. Similarly, the impact of the disease in these high-risk populations can influence vaccination priority [5].

A recent study in Peru found similar results in the general population compared with those we found in Colombia among physicians [25]. In a survey including 17,162 adults, the vaccination intention was 74.9%, higher in the capital province Lima, 81.4% [25]. Our study did not find significant differences by region, but it was also higher in the capital city, Bogotá (93.6%). Recently, in Greek health professionals, a similar acceptance level for the COVID-19 vaccine was found (78.5%) [26]. Additionally, in Spain, a study among 731 participants found that 77.6% of them intend to be vaccinated against COVID-19 [27]. This study included different healthcare professionals [27].

The current key goal in most countries is to vaccinate rapidly. As fast as possible, the herd immunity is reached, better to halt the multiple effects of the COVID-19 pandemic. Recently, studies assessing the impact of COVID-19 vaccines, as is the case of the Pfizer-BioNTech BNT162b2 COVID-19 vaccine, in countries such as Israel, have shown the effectiveness of this intervention at the population level as a consequence of their nationwide mass vaccination [28]. In addition to vaccine hesitance by the population, many challenges are faced to reach herd immunity. That includes the emergence of the new variants of concern (VOC) that may potentially affect the efficacy of the vaccines, as has been suggested, and deserve specific assessments on each of the major VOC currently circulating in the world, e.g., 501Y.V1 (B.1.1.7), 501Y.V2 (B.1.351), P.1 (501Y.V3) [29]. In South Africa, the AstraZeneca-Oxford (Cambridge, UK) vaccine would be affected by the 501Y.V2 (B.1.351) VOC [30]. Fortunately, recent data suggest that Novavax (Gaithersburg, MD, USA) vaccine efficacy is 86% against the United Kingdom variant, 501Y.V1 (B.1.1.7), and 60% against the South African variant [31]. Finally, for the BNT162b2 vaccine, recent data suggest that the neutralization of SARS-CoV-2 variants or corresponding pseudo viruses by convalescent or post-immunization sera against the virus with three mutations from the South Africa variant (E484K + N501Y + D614G) was slightly lower than the neutralization against the N501Y virus or the virus with three mutations from the United Kingdom variant. Nevertheless, multiple studies are ongoing to assess the clinical data and weight more robust interpretations [32]. This will be also important for Colombia, which confirmed the arrival of P.1 (501Y.V3) VOC in Bogota on 12 March 2021.

## 5. Limitations

This study’s main strength is that it is the first to be carried out in Colombian territory and medical personnel. One of the limitations is that it did not embrace comprehensive individual vaccination history in the last decade or if they had a relative or acquaintance with COVID-19 and the participants’ socioeconomic level. However, this was theoretically explored (Figure 3) as to what could have been the assumption of confounding bias we could not control.

## 6. Conclusions

Globally, there are still multiple challenges in the control of COVID-19 [33,34]. Vaccination is a critical tool for the integrated control of this deadly emerging disease [35,36], particularly amongst healthcare workers, a risk population that has been significantly impacted, particularly in Latin America and Colombia [37,38]. There is a high perception of the intention to vaccinate doctors in Colombia against COVID-19. But it is very similar to that of the general population, at least based on data reported in other studies. This intention supports the community’s perception and disposition to be vaccinated at the time of vaccine availability. As a tool to halt the epidemic in a country significantly affected by COVID-19, over 2.3 million cases have been reported to date.

## Figures and Tables

**Table 1:** Characteristics of the population according to the acceptance of free vaccination with an effectiveness of 60% and 80%.

Characteristics	60% Effectiveness		*p*-Values	80% Effectiveness		*p*-Values
	No (n = 245, 23.0%)	Yes (n = 821, 77.0%)		No (n = 99, 9.3%)	Yes (n = 967, 90.7%)	
Age, mean (years, ±SD)	47.4 (18)	45.1 (19)	0.003 ^¥^	48.7 (20)	45.3 (19)	0.004 ^¥^
Gender						
Male	440 (78.2)	123 (21.8)	0.031 ^‡^	44 (7.8)	519 (92.2)	0.187 ^‡^
Female	380 (75.8)	121 (24.2)		55 (11.0)	446 (89.0)	
Indeterminate	1 (50.0)	1 (50.0)		0 (0.0)	2 (100.0)	0.024
Number of years of graduate (±SD)	19.6 (19)	17.8 (19)	0.0312 ^¥^	20.8 (20)	17.9 (19)	0.0248 ^¥^
Specialty						
Pediatrics	48 (14.8)	277 (85.2)	<0.0001 ^†^	17 (5.2)	308 (94.8)	0.021 ^†^
General medicine	22 (24.2)	69 (75.8)		7 (7.7)	84 (92.3)	
Surgical	98 (25.3)	289 (74.7)		42 (10.9)	345 (89.1)	
Clinics	61 (27.2)	163 (72.8)		27 (12.1)	197 (87.9)	
Administrative	16 (41.0)	23 (59.0)		6 (15.4)	33 (84.6)	
Work performance area						
External consultation	99 (23.8)	317 (76.2)	0.004 ^†^	42 (10.1)	374 (89.9)	0.005 ^†^
Critical or intermediate care (adult/pediatric)	41 (22.4)	142 (77.6)		15 (8.2)	168 (91.8)	
Emergencies	39 (23.4)	128 (76.6)		12 (7.2)	155 (92.8)	
Hospitalization	21 (13.0)	140 (87.0)		8 (5.0)	153 (95.0)	
Administrative	8 (25.0)	24 (75.0)		2 (6.3)	30 (93.8)	
Other	37 (34.6)	70 (65.4)		20 (18.7)	87 (81.3)	
Department where you currently work						
Caribbean coast	53 (29.0)	130 (71.0)	0.180 ^†^	20 (10.9)	163 (89.1)	0.302 ^†^
East	49 (24.0)	155 (76.0)		21 (10.3)	183 (89.7)	
Bogotá D.C (Capital)	58 (18.6)	253 (81.4)		20 (6.4)	291 (93.6)	
Pacific Coast	30 (24.8)	91 (75.2)		16 (13.2)	105 (86.8)	
Center (Antioquia, coffee region)	45 (22.8)	152 (77.2)		19 (9.6)	178 (90.4)	
Plains (Meta, Arauca, Caquetá, Casanare)	7 (19.4)	29 (80.6)		3 (8.3)	33 (91.7)	
Do you have teaching functions?						
No	127 (22.5)	437 (77.5)	0.702 ^‡^	47 (8.3)	517 (91.7)	0.255 ^‡^
Yes	118 (23.5)	384 (76.5)		52 (10.4)	450 (89.6)	
Have you carried out research projects that have generated the publication of articles or conference papers?						
No	106 (22.9)	356 (77.1)	0.979 ^‡^	37 (8.0)	425 (92.0)	0.209 ^‡^
Yes	139 (23.0)	465 (77.0)		62 (10.3)	542 (89.7)	
The number of patients seen per day.	16.3 (10)	17.1 (10)	0.371 ^¥^	15.7 (14)	17.0 (10)	0.295 ^¥^
Do you know someone with a confirmed positive diagnosis for COVID-19						
No	4 (11.8)	30 (88.2)	0.114 ^‡^	1 (2.9)	33 (97.1)	0.195 ^‡^
Yes	241 (23.4)	791 (76.6)		98 (9.5)	934 (90.5)	
Do you know anyone who has died from COVID-19?						
No	32 (18.1)	145 (81.9)	0.089 ^‡^	16 (9.0)	161 (91.0)	0.901 ^‡^
Yes	213 (24.0)	676 (76.0)		83 (9.3)	806 (90.7)	
Do you know any person who has had a positive diagnosis for COVID-19 who has not died?						
No	2 (8.3)	22 (91.7)	0.084 ^‡^	0 (0.0)	24 (100.0)	0.113 ^‡^
Yes	243 (23.3)	799 (76.7)		99 (9.5)	943 (90.5)	
How many people live with you (who eat and sleep in the same house)?						
0	17 (21.0)	64 (79.0)	0.079 ^†^	11 (13.6)	70 (86.4)	0.243 ^†^
1	37 (19.4)	154 (80.6)		17 (8.9)	174 (91.1)	
2	56 (30.9)	125 (69.1)		22 (12.2)	159 (87.8)	
3	61 (22.1)	215 (77.9)		25 (9.1)	251 (90.9)	
4 and more	74 (22.0)	263 (78.0)		24 (7.1)	313 (92.9)	
How many children do you have?						
0	5 (12.5)	35 (87.5)	0.087 ^†^	0 (0.0)	40 (100.0)	0.255 ^†^
1	10 (19.2)	42 (80.8)		5 (9.6)	47 (90.4)	
2	35 (20.7)	134 (79.3)		12 (7.1)	157 (92.9)	
3 and more	24 (31.6)	52 (68.4)		7 (9.2)	69 (90.8)	
How many people over 70 years of age live with you (who sleep and eat in the same house)?						
0	202 (22.9)	682 (77.1)	0.973 ^†^	80 (9.0)	804 (91.0)	0.557 ^†^
1	33 (23.7)	106 (76.3)		13 (9.4)	126 (90.6)	
Two and more	10 (23.3)	33 (76.7)		6 (14.0)	37 (86.0)	
Do you live with someone with at least one of the following comorbidities: Diabetes, Hypertension, Heart disease, Congenital malformations, cancer, immunosuppression, obesity?						
No	155 (23.2)	512 (76.8)	0.798 ^‡^	66 (9.9)	601 (90.1)	0.377 ^‡^
Yes	90 (22.6)	309 (77.4)		33 (8.3)	366 (91.7)	
Do you suffer from any comorbidity?						
No	158 (22.9)	531 (77.1)	0.957 ^‡^	70 (10.2)	619 (89.8)	0.185 ^‡^
Yes	87 (23.1)	290 (76.9)		29 (7.7)	348 (92.3)	
The answer was yes; which comorbidity?						
No	13 (31.7)	28 (68.3)	0.494 ^†^	5 (12.2)	36 (87.8)	0.820 ^†^
Hypertension	7 (36.8)	12 (63.2)		1 (5.3)	18 (94.7)	
Obesity	1 (100.0)	0 (0.0)		0 (0.0)	1 (100.0)	
Diabetes	1 (16.7)	5 (83.3)		0 (0.0)	6 (100.0)	
Other	2 (22.2)	7 (77.8)		1 (11.1)	8 (88.9)	
Have you ever paid for a vaccine?						
No	56 (34.1)	108 (65.9)	<0.0001 ^‡^	33 (20.1)	131 (79.9)	<0.0001 ^‡^
Yes	189 (21.0)	713 (79.0)		66 (7.3)	836 (92.7)	
You would recommend that your parents or people over 70 years get the COVID-19 vaccine, if available.						
No	96 (79.3)	25 (20.7)	<0.0001 ^‡^	68 (56.2)	53 (43.8)	<0.0001 ^‡^
Yes	149 (15.8)	796 (84.2)		31 (3.3)	914 (96.7)	
You would give your children the vaccine for COVID-19, if available						
No	115 (75.7)	37 (24.3)	<0.0001 ^‡^	78 (51.3)	74 (48.7)	<0.0001 ^‡^
Yes	130 (14.2)	784 (85.8)		21 (2.3)	893 (97.7)	

^‡^: *p*-value determined by Chi2 test. ^¥^: *p*-value determined by the Mann-Whitney U test. ^†^: *p*-value determined by Fisher’s exact test. SD: Standard deviation.

**Table 2:** Variables associated with the acceptance of free vaccination with an effectiveness of 60%.

Characteristics	Crude Model			Fitted Model *		
	PR	95%CI	*p*	PR	95%CI	*p*
Medical specialty						
Administrative	Ref.			Ref.		
General medicine	2.18	0.98–4.84	0.055	1.91	0.85–4.28	0.114
Surgical	2.05	1.04–4.04	0.038	2.29	1.15–4.56	0.018
Clinics	1.85	0.92–3.75	0.084	4.48	0.98–4.04	0.057
Pediatrics	4.01	1.97–8.14	<0.0001	0.22	2.19–9.16	<0.0001
Department where you currently work						
Caribbean coast	Ref.			Ref.		
East	1.28	0.81–2.02	0.271	1.26	0.79–1.99	0.317
Bogotá D.C (Capital)	1.77	1.15–2.72	0.008	1.86	1.20–2.88	0.005
Pacific Coast	1.23	0.73–2.08	0.425	1.28	0.76–2.18	0.344
Center (Antioquia, coffee region)	1.37	0.86–2.18	0.174	1.48	0.93–2.37	0.096
Plains (Meta, Arauca, Caquetá, Casanare)	1.68	0.69–4.09	0.246	1.69	0.69–4.12	0.243
How many children do you have?						
0	Ref.			Ref.		
1	0.6	0.18–1.92	0.389	0.58	0.17–1.97	0.386
2	0.54	0.19–1.49	0.241	0.51	0.17–1.57	0.247
3 and more	0.30	0.10–0.88	0.029	0.27	0.08–0.95	0.043
Have you ever paid for a vaccine?						
No	Ref.			Ref.		
Yes	1.95	1.36–2.80	<0.0001	1.83	1.27–2.65	0.001
You would recommend that your parents or people over 70 years get the COVID-19 vaccine, if available.						
No	Ref.			Ref.		
Yes	20.51	12.7–32.9	<0.0001	21.8	13.4–35.2	<0.0001
You would give your children the vaccine for COVID-19, if available						
No	Ref.			Ref.		
Yes	18.7	12.3–28.3	<0.0001	20.5	13.4–31.5	<0.0001

* Model Adjusted for gender, age. PR: prevalence ratio. 95%CI: 95% confidence interval; *p*: *p*-value; Ref: reference category.

**Table 3:** Variables associated with the acceptance of free vaccination with an effectiveness of 80%.

Characteristics	Crude Model			Fitted Model *		
	PR	95%CI	*p*	PR	95%CI	*p*
Medical specialty						
Administrative	Ref.			Ref.		
General medicine	2.18	0.68–6.97	0.188	1.88	0.58–6.06	0.290
Surgical	1.49	0.59–3.77	0.396	1.76	0.68–4.51	0.238
Clinics	1.32	0.50–3.45	0.563	1.47	0.56–3.88	0.428
Pediatrics	3.29	1.21–8.93	0.019	3.72	1.36–10.20	0.010
Have you ever paid for a vaccine?						
No	Ref.			Ref.		
Yes	3.19	2.02–5.03	<0.0001	2.91	1.83–4.64	<0.0001
You would recommend that your parents or people over 70 years get the COVID-19 vaccine, if available.						
No	Ref.			Ref.		
Yes	37.8	22.7–62.8	<0.0001	44.3	25.8–75.9	<0.0001
You would give your children the vaccine for COVID-19, if available						
No	Ref.			Ref.		
Yes	44.4	26.1–76.6	<0.0001	55.8	31.3–99.3	<0.0001

* Model Adjusted for gender, age. PR: prevalence ratio. 95%CI: 95% confidence interval; *p*: *p*-value; Ref: reference category.

## Data Availability

The data presented in this study are available on request from the corresponding author.
